# Dramatic Devices?: Medical Procedures May Expose Infants to BPA

**DOI:** 10.1289/ehp.117-a163a

**Published:** 2009-04

**Authors:** Julia R. Barrett

The industrial chemical bisphenol A (BPA) is widely used to make polycarbonate plastic and epoxy resins. Low-level exposure to BPA has been shown to cause endocrine disruption in animal experiments, resulting in abnormal development of the prostate and mammary glands, among other adverse outcomes. Interpreting these studies with regard to human health has generated substantial debate, one heightened by growing awareness of the widespread nature of BPA exposure. In this report, researchers describe evidence of substantial exposure to BPA and other potential endocrine disruptors through medical treatment of premature infants **[*EHP*** **117:639–644; Calafat et al.]**.

In an earlier study by the same group [*EHP* 113:1222–1225 (2005)], urine samples were collected from 54 premature infants in neonatal intensive care units at two institutions. The infants required medical interventions such as ventilation, enteral feeding, parenteral feeding, and indwelling catheterization. Some of the medical devices used in these procedures contained the plasticizer di(2-ethylhexyl) phthalate (DEHP), and urine sample analysis revealed that concentrations of DEHP metabolites correlated with the relative intensity (low, medium, or high) of medical device use—that is, the variety, invasiveness, and duration of the procedure(s) that each infant underwent.

In the current study, the team used some of those same urine samples to assess exposure to several other potential endocrine disruptors, including BPA, the antimicrobial triclosan, the preservatives methyl paraben and propyl paraben (found in personal care products), and benzophenone-3, a sunscreen agent. For each chemical, urinary concentrations of the free (unmetabolized) and total (both free and conjugated, or metabolized) compounds were measured.

The detection of BPA and both parabens in the urine of all the samples analyzed suggested that all the infants had been exposed to those chemicals. Benzophenone-3 was detected in the urine of all but 2 infants, whereas triclosan was detected in only 8 infants. Urinary concentrations of BPA correlated with those of DEHP, suggesting a common pathway of exposure. Of the chemicals assessed in the current study, only BPA correlated significantly with the intensity of medical device use, although the authors have no information about whether or how BPA is used in these devices.

The median urinary BPA concentration in these infants was almost 10 times higher than levels reported elsewhere for 6- to 11-year-old children in the general population. The fact that more than 90% of the BPA was conjugated suggests that premature infants are able to metabolize the compound even though metabolic pathways typically do not function at an adult level for some months after birth. The authors suggest that, given concerns over BPA toxicity and the demonstrated exposure, use of BPA-free products may be justified in this developmentally vulnerable population.

